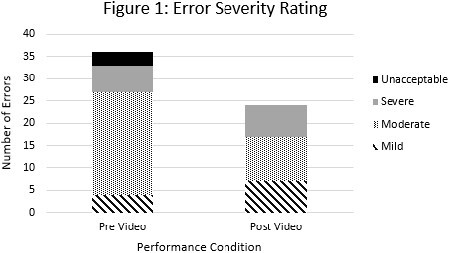# 753 Implementation of a QR-code-linked Instructional Video for Resting Hand Splint Application by Non-therapists

**DOI:** 10.1093/jbcr/irae036.295

**Published:** 2024-04-17

**Authors:** Tonya S Terken, Kristine A Parker, Gretchen J Carrougher, Paige L MacKenzie, Dylan S Jason, Tam N Pham

**Affiliations:** Harborview Medical Center, Seattle, WA; University of Washington Regional Burn Center at Harborview, Seattle, WA; UW Medicine Regional Burn Center, Seattle, WA; UW Medicine Regional Burn Center at Harborview, Seattle, WA; University of Washington - Harborview Burn Center, Seattle, WA; Harborview Medical Center, Seattle, WA; University of Washington Regional Burn Center at Harborview, Seattle, WA; UW Medicine Regional Burn Center, Seattle, WA; UW Medicine Regional Burn Center at Harborview, Seattle, WA; University of Washington - Harborview Burn Center, Seattle, WA; Harborview Medical Center, Seattle, WA; University of Washington Regional Burn Center at Harborview, Seattle, WA; UW Medicine Regional Burn Center, Seattle, WA; UW Medicine Regional Burn Center at Harborview, Seattle, WA; University of Washington - Harborview Burn Center, Seattle, WA; Harborview Medical Center, Seattle, WA; University of Washington Regional Burn Center at Harborview, Seattle, WA; UW Medicine Regional Burn Center, Seattle, WA; UW Medicine Regional Burn Center at Harborview, Seattle, WA; University of Washington - Harborview Burn Center, Seattle, WA; Harborview Medical Center, Seattle, WA; University of Washington Regional Burn Center at Harborview, Seattle, WA; UW Medicine Regional Burn Center, Seattle, WA; UW Medicine Regional Burn Center at Harborview, Seattle, WA; University of Washington - Harborview Burn Center, Seattle, WA; Harborview Medical Center, Seattle, WA; University of Washington Regional Burn Center at Harborview, Seattle, WA; UW Medicine Regional Burn Center, Seattle, WA; UW Medicine Regional Burn Center at Harborview, Seattle, WA; University of Washington - Harborview Burn Center, Seattle, WA

## Abstract

**Introduction:**

Custom-made rigid orthotics such as a resting hand splints are a cornerstone of burn rehabilitation. Non-therapists are often asked to apply or reapply these specialized splints. Improper application not only reduces their efficacy but may also risk harm, such as pressure ulceration. This quality improvement project aims to improve resting hand splint application via a short instructional video accessible by QR code placed directly on the splint.

**Methods:**

Phase I consisted of directly observing non-therapists in resting hand splint application, which informed the development of a < 2-minute video demonstrating correct application. Suboptimal techniques were compiled and rated on a 4-point error severity scale by 3 therapists. Phase II queried providers who reviewed the initial draft video and informed the team on finalizing the instructional video. Phase III determined video effectiveness: 1) by comparing application techniques before and after watching the video, and 2) by tracking of QR code usage.

**Results:**

Phase I assessed 9 clinicians for splint application. All trials contained application errors, (mean 4 errors/trial, range 2-8, with 87% agreement between 2 trained observers). In Phase II, 8 clinicians evaluated the draft video: 100% reported that the content was clear; with ideal video length reported as < 2 to 5 minutes; and video speed and length were deemed “just right”. In Phase III, viewing the video resulted in fewer applications errors and shifted toward milder error types (Figure 1). Additional Phase III assessments are ongoing.

**Conclusions:**

A short instructional video improves application accuracy by providers less familiar with resting hand splint application. A QR-code that links to a short instruction video is a useful strategy for delivery of just-in-time education for non-therapists.

**Applicability of Research to Practice:**

Just-in-time instructional videos facilitate burn rehabilitation tasks for non-therapists. This strategy can potentially expand to patients and caregivers as well.